# Engineered Biomaterials Trigger Remineralization and Antimicrobial Effects for Dental Caries Restoration

**DOI:** 10.3390/molecules28176373

**Published:** 2023-08-31

**Authors:** Yuexiao Li, Minda Liu, Mingyu Xue, Yuanyuan Kang, Dongjuan Liu, Yan Wen, Duoyi Zhao, Boyu Guan

**Affiliations:** 1 Department of Oral and Maxillofacial Surgery, School and Hospital of Stomatology, China Medical University, Shenyang 110022, China; 2 School and Hospital of Stomatology, China Medical University, Shenyang 110002, China; 3 Department of Orthopedics, The Fourth Affiliated Hospital of China Medical University, Shenyang 110032, China

**Keywords:** dental caries, biomaterials, dentin remineralization, antibacterial

## Abstract

Dental caries is the most prevalent chronic disease globally, significantly impacting individuals’ quality of life. A key reason behind the failure of implanted restorations is their biological inactivity, meaning they are unable to form crosslinks with the surrounding tooth structures, thus making patients susceptible to implant loss and recurrent tooth decay. For the treatment of caries, antibacterial medicine and remineralization are effective means of treating the recurrence of caries. Owing to the rapid progression in the biomaterials field, several biomaterials have been reported to display antimicrobial properties and aid in dentin remineralization. Bioactive materials hold considerable potential in diminishing biofilm accumulation, inhibiting the process of demineralization, enabling dentin remineralization, and combating bacteria related to caries. Bioactive materials, such as fluoride, amorphous calcium phosphate, bioactive glass, collagen, and resin-based materials, have demonstrated their effectiveness in promoting dentin remineralization and exerting antibacterial effects on dental caries. However, the concentration of fluoride needs to be strictly controlled. Although amorphous calcium phosphate can provide the necessary calcium and phosphorus ions for remineralization, it falls short in delivering the mechanical strength required for oral mastication. Resin-based materials also offer different advantages due to the complexity of their design. In this review, we delve into the application of advanced bioactive materials for enhancing dentin remineralization and antibacterial properties. We eagerly anticipate future developments in bioactive materials for the treatment of dental caries.

## 1. Introduction

Dental caries, commonly known as tooth decay, is one of the most widespread chronic diseases globally, resulting from the local destruction of susceptible teeth by acids produced by bacterial fermentation in food [1]. Dental caries not only affects patients aesthetically but also significantly impacts patients’ oral health, often leading to difficulties in chewing, reduced appetite, weight loss, sleep disorders, behavior changes, and other manifestations [2]. Approximately 621 million children reportedly suffer from dental caries globally [3]. The prevalence of dental caries in adults and the elderly is progressively increasing [4]. Nearly 91% of adults suffer from dental caries, and the incidence can reach 98% in the elderly [5]. The financial burden of oral diseases is substantial, with dental caries ranking as the most prevalent condition in the 2010 global burden of disease report [4]. In the United States alone, annual spending on dental diseases amounts to USD 37.047 billion [6].

The consumption of carbohydrates and the subsequent acidic environment resulting from their fermentation foster the growth of acid-tolerant bacteria. Once caries bacteria proliferate, they form a plaque biofilm, leading to loss of tooth structure and, in severe cases, potential infection and tooth loss [7]. Numerous filling materials are currently used clinically to restore dental defects caused by caries, such as bioceramics, hydroxyapatite, resin restorations, and amalgam restorations [8,9]. Amalgam restorations remain the material of choice for restoration in some low- and middle-income countries [10]. However, the plaque that develops at the interface of dental fillings often triggers dentin hypersensitivity reactions, leading to secondary caries [11]. Studies have indicated that 36.5% of filling failures are attributed to secondary dental caries [12]. Ideal fillings should provide mechanical strength to the defect while supporting antimicrobial action and tooth remineralization conditions.

Dental caries results from an imbalance between demineralization and remineralization processes in oral bacterial biofilms and tooth minerals. Demineralization involves the net loss of calcium and phosphate ions from the tooth structure, while remineralization refers to adding calcium and phosphate back to the enamel from supersaturated oral fluid [13]. Supersaturated calcium and phosphate solutions and fluoride solutions are commonly used remineralizing agents to promote dental caries remineralization [14]. Although these solutions can encourage enamel remineralization and reduce the incidence of caries, they have minimal effects on dentin remineralization [15]. Dentin, as a complex part of the tooth, comprises an organic matrix of 70% hydroxyapatite (HAP), 20% type I collagen, and 10% water [16]. The remineralization of dentin involves the regeneration of the mineralized collagen matrix and the formation of hydroxyapatite crystals [17]. Fluoride is the cornerstone of tooth remineralization. Higher concentrations of fluoride are associated with optimal caries control [18]. However, fluoride is also considered a neurotoxin, raising concerns about its safety [17].

Non-fluoride-induced dentin remineralization has emerged as a new direction in dental restoration. Biomimetic medicine has made significant progress in biomedicine. Current research uses tissue engineering methods to remineralize lost minerals into the structure [19]. Amorphous calcium phosphate (ACP) shows promise in providing sufficient calcium and phosphorus ions for caries remineralization and in promoting dentin remineralization. However, due to its low mechanical strength, ACP falls short of meeting the masticatory demands of teeth. Collagen, a vital component of the dentin matrix, offers crystalline binding sites for dentin remineralization. However, oral bacteria quickly degrade collagen in an acidic mouth environment. Synthetic self-assembled polypeptides exhibit antibacterial effects around the teeth but involve complex preparation methods.

Resin-based materials have recently proven to be effective as fillings for the treatment of dental caries. Resin-based materials have significantly improved dentin remineralization and antimicrobial properties by enhancing the preparation method and raw material ratios. Additionally, in the preclinical research stage, several polymer materials for filling defects have been developed (Table 1 and Scheme diagram).

This review explores the application of biologically active biomaterials to enhance remineralization, antimicrobial effects, and future research prospects in treating dental caries.

## 2. Fluoride

The dentin surrounding has a relatively high pH of approximately 6.2 [46]. Environments with lower pH levels can cause demineralization of teeth. The classical remineralization pathway is based on the ionic crystallization pathway, where primary ions or molecules form clusters of nuclei that grow into lattice-sized structures to compensate for defects [47]. In contrast to the classical mechanism of single-crystal growth mediated by atoms/molecules, non-classical crystallization involves particle-mediated growth and assembly mechanisms coupled with existing processes such as oriented attachment and mesocrystal formation. This pathway, known as “non-classical crystallization,” relies on an exposed organic matrix for bottom-up remineralization [48]. ACP nanoparticles penetrate the collagen matrix and self-assemble in a crystal phase to achieve dentin remineralization [11]. Demineralization and remineralization are reversible processes.

When the ambient pH exceeds 7.0, the excess calcium and phosphate ions on the surface of the mouth adhere to the tooth in an amorphous form. However, the oral cavity’s variable environment may not provide sufficient conditions for tooth remineralization. Among various compounds, fluoride remains the gold standard for inhibiting demineralization [49]. Fluoride directly hinders bacterial metabolism in biofilms and remineralizes decalcified teeth by reducing enamel solubility by forming fluorapatite at low pH [50,51]. The ability of fluoride to enhance early caries in mineralization has been demonstrated [52]. However, the effectiveness of fluoride in tooth remineralization depends on the fluoride ion concentration [53]. High concentrations of fluoride ions can lead to undesirable consequences. Furthermore, the application of fluoride in recent years has resulted in oral bacteria developing resistance to fluoride [54]. Consequently, there is an urgent need to improve fluoride treatments to combat fluoride-resistant oral bacteria.

Arginine, a naturally occurring amino acid, raises the pH of biofilms, consequently limiting their formation [55]. Arginine interacts with sodium fluoride at low concentrations (500 ppm) to eliminate mutant streptococci, thus bolstering anti-caries effects. In an innovative approach, Mohammed et al. blended 2% arginine with a toothpaste rich in sodium fluoride in a 1:3 ratio, producing a novel cleaning toothpaste [20]. The distinct advantage of this toothpaste lies in its use of arginine’s positively charged guanidine groups and negatively charged fluoride ions, which serve to augment the fluoride ion concentration in the toothpaste, thereby boosting its remineralization potential. However, a rise in arginine concentration inversely impacts the pH of the toothpaste, potentially undermining tooth remineralization. Furthermore, toothpaste fails to maintain a persistent environment conducive to remineralization.

Polyhexamethylene biguanide (PHMB), a cationic antimicrobial agent with amphiphilic properties, exhibits wide-ranging antibacterial features and optimal biosafety. Chen et al., aiming to increase fluoride concentration, incrementally added an AgF solution to an PHMB solution, which resulted in a fluorinated cationic polymer known as PHMB-F [21]. Compared with PHMB, PHMB-F possesses a less dense structure, tinier voids, and superior surface hardness. Additionally, in vitro studies have demonstrated that PHMB-F displays enhanced antibacterial effectiveness. Although PHMB-F’s antibacterial and remineralization competencies are promising, its long-term in vivo safety remains uncertain. Fluoride has an observable preventive impact on enamel but is less effective in conspicuous caries lesions [56].

## 3. Amorphous Calcium Phosphate

Amorphous calcium phosphate (ACP) is produced in aqueous solutions supersaturated with calcium ions and phosphates, demonstrating significant biocompatibility and bioactivity [57]. ACP transforms hydroxyapatite (HA) in the presence of water [58]. However, the complexity of controlling the ACP’s morphology and structure hinders its widespread use across various fields. ACP provides an external source of calcium and phosphorus ions for the remineralization of caries, regardless of the availability of these ions in saliva. A notable advantage of nano-amorphous calcium phosphate (NACP) is its increased ion release at pH 4, which minimizes enamel demineralization, and in turn, caries formation [59].

Shen et al. introduced a single-step, microwave-assisted solvothermal technique to synthesize sticky silver-calcium phosphate (GSCP) for dental antimicrobial use and remineralization [22]. Adenosine triphosphate (ATP) aids GSCP formation by stabilizing the ACP phase and binding sites for Ag. The release of calcium ions and phosphates from GSCP sets the groundwork for remineralization and disrupts bacterial growth by inactivating bacterial respiratory enzymes. GSCP forms a consistent layer on the dentin surface, providing efficient dentin tubule coverage. However, due to its jelly-like structure, GSCP lacks stable adhesion to the tooth surface. Therefore, acid-resistant adhesives with remineralization properties could be superior biomaterials for the prevention of caries.

Fan et al. combined ACP with a binder to create nanoparticles [23]. The average size of these NACP particles was 116 nm. Different combinations of ACP and binders resulted in nanoparticles with varied bond strengths. Their research showed that nanoparticles composed of 35% NACP achieved a bond strength of 11.1 ± 3.8 Mpa, ensuring nanoparticle stability. NACP released more calcium ions and phosphates than ACP, substantially elevating calcium and phosphorus levels in biofilms. After a 7-day immersion in artificial saliva, tooth enamel treated with the ACP binder demonstrated superior remineralization compared to ACP, achieving a remineralization value of 52.29 ± 4.79%. The presence of ACP in the binder also lowered lactic acid production in biofilms, aiding in tooth remineralization. However, ACP adhesives have a downside: the released calcium and phosphorus ions have only been proven effective in vitro, with their in vivo effectiveness remaining uncertain.

To increase the reduction and remineralization capabilities of biofilm bacteria, Patteera et al. refined an ACP adhesive using polyethylene glycol as a dispersant, ethyl alcohol as a carrier, and α-mangiferin as an antimicrobial agent [60]. Modifying the formulation ratio could simultaneously enhance the acid-resistant activity of tooth enamel and retain its remineralization capacity.

Casein phosphopeptide is known to supersaturate saliva with calcium, phosphate, and fluoride ions, aiding in the remineralization of mineral-deficient tooth structures [61]. The topical application of fluoride-containing amorphous calcium phosphate integrated with casein phosphopeptide (CPP-ACP/F) has shown plaque removal effectiveness in children [62]. However, although CPP-ACP is widely used clinically for tooth surface remineralization, the mineralized layer on the CPP-ACP surface has been found to age significantly and peel off after thermal cycling [58].

Sodium trimetaphosphate has been proven to lessen hydroxyapatite (HA) solubility and mineral exchange, disrupt the demineralization process, and lower the occurrence of dental caries [63]. Marcelle et al. introduced sodium trimetaphosphate to CPP-ACP to enhance its remineralization capacity [24]. After a 6 h treatment with the modified CPP-ACP, a reduction in mineral loss on the tooth surface was observed, suggesting that the modified CPP-ACP can diminish tooth demineralization.

In a different study, James et al. incorporated stannous fluoride (SnF2) into CPP-ACP to form SnF2-CPP-ACP [25]. Sn interacts with the tooth surface to augment hardness and prevent the exposure of calcium to the enamel surface, increasing its resistance to demineralization [64]. SnF2 achieves dentin remineralization by replacing hydroxyapatite (HAp) in the tooth enamel to form fluorinated apatite (FAp) [65]. In vitro simulations found that SnF2 + CPP-ACP could enhance remineralization by 32% on the tenth day compared to CPP-ACP. Additionally, the inclusion of SnF2 also strengthens the mixture’s ability to release fluoride ions under acidic conditions.

**Figure 1:**
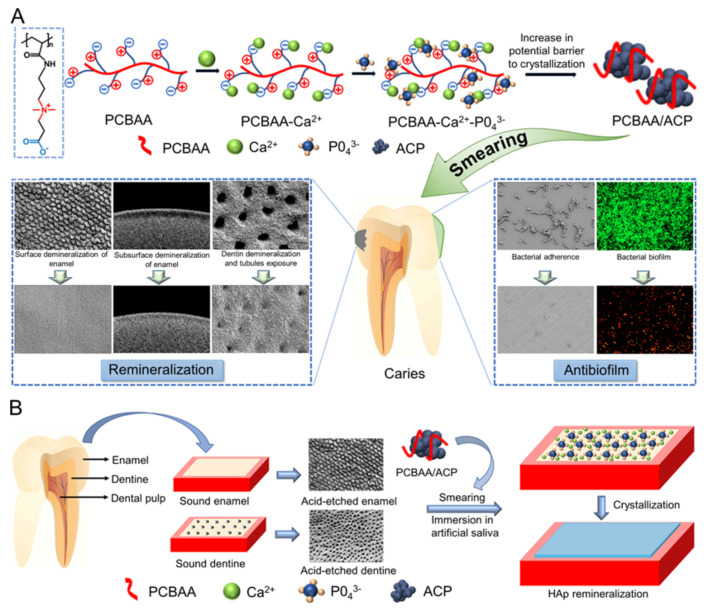
(**A**) Schematic illustration of the PCBAA/ACP nanocomposite with dual antibiofilm and remineralization functions. Evaluation of the effect of the PCBAA/ACP nanocomposite on inhibiting cariogenic bacterial adhesion and biofilm formation on the enamel surface and promoting enamel remineralization and DT occlusion. (**B**) Schematic demonstration of the in vitro remineralization of enamel and dentin with the PCBAA/ACP nanocomposite. Reproduced with permission from [26].

Similar to SnF2, silver diamine fluoride (SDF) is utilized as an alternate treatment for caries prevention [66]. Although fluoride has demonstrated some effectiveness when combined with ACP, its cytotoxicity should not be overlooked. A zwitterionic compound, poly (carboxybetaine acrylamide) (PCBAA), was developed to stabilize ACP, forming PCBAA/ACP nanocomposites (Figure 1A) [26]. PCBAA provides more cations to eliminate bacteria in biofilms under acidic conditions. The PCBAA/ACP nanocomposite, with an average particle size of 50.67 ± 2.37 nm, displays high calcium ion release efficiency. In vitro tests showed that PCBAA/ACP nanocomposites could kill 90.4% of bacteria and inhibit bacterial biofilm formation within six hours. Another advantage of PCBAA/ACP nanocomposites is that their small particle size allows for efficient penetration into the dentin tubules for deep remineralization (Figure 1B). Although the crystals formed in the repair layer of PCBAA/ACP nanocomposites are not enamel-like, their mechanical properties and stability significantly surpass those achieved in traditional fluoride therapy.

## 4. Bioactive Glass

Bioactive glass (BAG) can interact with hard tissues, forming a calcium phosphate-rich layer that promotes the remineralization of both tooth enamel and dentin [67]. BAG has shown superior recovery abilities in the face of demineralizing and remineralizing solutions, with low concentrations capable of restoring surface hardness [68]. Researchers have found that dentin remineralization does not occur through spontaneous precipitation or mineral nucleation on the organic substrate, but rather, through the growth of residual crystals in the lesion [69]. Dentin remineralization can still occur despite mineral loss, provided no bacterial invasion exists [70]. Under these conditions, it becomes crucial to seal the dentin tubules. It has been reported that BAG can penetrate these tubules to form a calcium phosphate layer, improving hardness and effectively treating dentin hypersensitivity [71].

The BAG particle size should be less than 20 μm for an optimum effect, as smaller particles tend to dissolve faster due to having a larger specific surface area [72]. Wu et al. compared the functions of 45S5 BAG, CPP-ACP, and sodium fluoride by simulating artificial dentin caries in vitro [27]. According to the AFM results, BAG primarily embeds in the dentin tubules to form deposits. Compared to the remineralization depth of CPP-ACP (111 ± 11 μm), BAG can facilitate deeper remineralization (165 ± 11 μm), proving more effective at sealing dentin tubules. The antibacterial effect of BAG can also be enhanced by incorporating zinc and silver ions.

Omar et al. integrated silver oxide into BAG to produce a BAG capable of delivering silver ions [28]. With an average particle size of 4–5 μm, this BAG can quickly enter the dentin tubules, reducing dentin hypersensitivity. The advantage of this BAG lies in replacing part of the silica with silver oxide. As silver ions are released, bridging oxygens are lost, further promoting the release of more cations. These ions are predominantly alkaline and can maintain the pH of their surroundings. Even though the BAG performs an antibacterial role by releasing silver ions, its disadvantage is that it diminishes the release of silicon ions.

Glass ionomer cement (GIC) is known for its antibacterial properties and adherence capabilities [73]. Adhesives enhance the graft’s attachment to the tooth, thus preventing the tooth from becoming loose and keeping bacterial invasion at bay. To increase the stability and wear resistance of GIC, Adriana et al. utilized composite resin (CR) [74]. The antibacterial effect on dentin can be enhanced by combining composites (CR + GIC) with silver diamine fluoride (SDF) treatment. To counteract the negative reactions triggered by high-silver ions in SDF, Adriana et al. utilized potassium iodide to neutralize silver ions, forming a silver iodide precipitate that could enhance dentin hardness. However, resin-modified GIC did not demonstrate superior remineralization capacity.

To augment the remineralization ability of BAG, Juliana et al. infused different concentrations of calcium fluoride into a pre-reacted glass ionomer (PRG) [29]. The average diameter of PRG was found to be 4.89 μm. Research has revealed that higher filler concentrations result in more released fluoride and calcium ions, leading to a stronger remineralization capability. Nevertheless, high calcium fluoride concentrations can impact the physical and chemical properties of the composites (calcium fluoride + PRG). When the weight of calcium fluoride exceeds 40% of the composite material, it can negatively affect its wear resistance and flexural strength. Moreover, if the weight of calcium fluoride surpasses 60%, it can also influence the solubility of the composite material.

In another study, the remineralization capacity of PRG filled with different concentrations of sodium fluoride was compared to that of 5% sodium fluoride [75]. It was found that PRG containing 40% sodium fluoride was more effective at tooth remineralization than 5% sodium fluoride filler. However, the 5% sodium fluoride filler could only show remineralization effects on the surface of the tooth. Achieving remineralization within the lesion can yield long-term results in the treatment of caries.

## 5. Collagen

The organic matrix of dentin, primarily comprising type I collagen, accounts for 30% of the total dentin weight [76]. The evolution of minimally invasive dentistry and the application of biomimetic remineralization technology to encourage the remineralization of demineralized dentin collagen have turned the reversal of dentin caries into a leading frontier in dentin caries prevention and treatment [77]. Research indicates that type I collagen positively influences the mineralization of apatite crystals by serving as a template or scaffold and offering binding sites [78]. Dentin remineralization capability decreased when collagenase was used to destroy dentin collagen, indirectly confirming the role of type I collagen in dentin remineralization [79].

**Figure 2:**
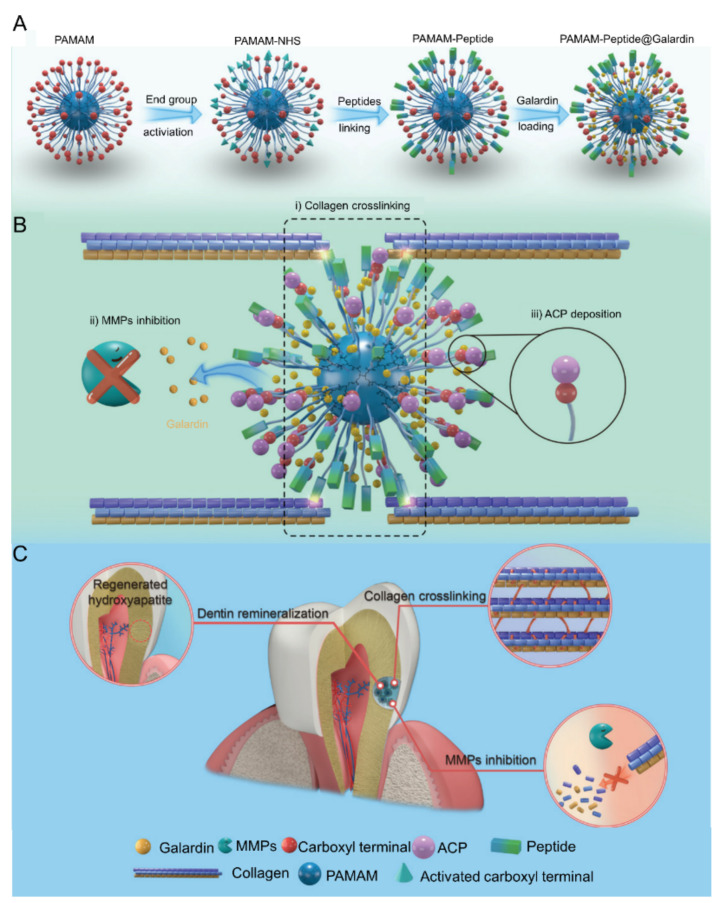
(**A**) Schematic illustration of the preparation of PAMAM-peptide@Galardin. (**B**) Schematic demonstration of the mechanisms of PAMAM-peptide@Galardin for dual collagen stabilization effects and remineralizing effect. (**C**) Schematic illustration of the dentin repair and dual collagen-protection effects by PAMAM-peptide@Galardin. Reproduced with permission from [30].

The oral acidic environment triggers matrix metalloproteinases (MMPs) to break down collagen [80]. Hence, MMP inhibitors can counteract collagen degradation and facilitate the remineralization process. Galardin, an MMP inhibitor, is insoluble in water, which restricts its clinical application [81]. Tao et al. integrated Galardin into the core of poly (amido amine) (PAMAM), with a peptide attached to the surface of PAMAM serving as a linker for collagen molecules (Figure 2) [30]. A key advantage of this polymer is that the carboxyl groups in PAMAM draw upon ACP to deposit on the collagen scaffold, enabling the biomimetic remineralization of dentin collagen. Concurrently, the release of Galardin inhibits MMPs, stabilizing collagen. The average particle size of PAMAM was 4.89 nm, while the average diameter of PAMAM-peptide was 16.8 nm. At pH 5.5, PAMAM-peptide continuously released Galardin, unloading 84.9% of Galardin within 120 h. In vitro tests showed that PAMAM-peptide offers superior collagen protection and induces remineralization more effectively than PAMAM. Furthermore, PAMAM-peptide can effectively combat dentin caries in rats.

To enhance PAMAM’s remineralization capability, Yang et al. combined ACP with PAMAM to create novel nanomaterials [31]. In vitro, remineralization models revealed that PAMAM/ACP demonstrates a superior capacity for human dentin type I collagen fibers, particularly intrafibrous remineralization.

Non-collagenous proteins (NCPs) and small biomolecules play crucial roles in intracollagen mineralization [82]. One such biomolecule, succinic acid (SA), has been widely researched for its ability to manage calcium mineral growth and as a bioactive molecule for complex tissue restoration [83]. In a study by Kim et al., SA was attached to a collagen fiber scaffold, thereby increasing the scaffold’s charge. This modification enhanced the attraction of calcium ions and improved the interaction between the collagen matrix and the amorphous precursor [32]. SA formed a stable bond with collagen fibers through hydrogen bonding. Collagen fiber scaffolds modified with SA were observed to accelerate the mineralization rate by 16 times in 6 h compared to those without SA modification. However, this modification did not significantly enhance the mechanical properties of the scaffold.

On another note, tannic acid (TA), a plant-based polyphenol, is known for its biostability and its potential for collagen crosslinking [84]. Studies have indicated that hydrogen bonds formed between collagen’s amide and TA’s hydroxyl group can strengthen the mechanical properties of dentin [84]. In one particular study, Kong et al. used TA and collagen to synthesize polymers, which were then used to induce dentin remineralization [33]. The crosslinking between TA and collagen reached 41.28%, resulting in a more compact polymer structure. This polymer exhibited increased resistance to collagenase, with a mass loss of only 3.84 ± 0.14% observed after five days in collagenase. In vitro, observations showed that the polymer could form a mineral layer of 3–4 μm within two days and achieve full remineralization in four days. The elasticity and hardness of the dentin post-remineralization were 19.1 ± 1.12 GPa and 0.68 ± 0.06 GPa, respectively, almost matching the strength of healthy dentin (21.7 ± 2.45 GPa and 0.9 ± 0.15 GPa). The impact of TA on dentin remineralization is well established, but the TA and polymer self-assembly processes are sensitive to variables such as pH, temperature, and TA concentration [85]. The details of the TA self-assembly process have not yet been comprehensively elucidated, calling for further study of these factors.

## 6. Self-Assembling Peptides

Dentin phosphorylated protein, the most prevalent non-collagen component found in dentin’s extracellular matrix, is known to act as a nucleation template for hydroxyapatite formation, thus initiating dentin mineralization [86]. Notably, the dentin phosphoprotein sequence contains a highly phosphorylated region rich in aspartate and serine (D-S-S), believed to exhibit a strong affinity towards calcium phosphate compounds [87]. Therefore, peptides that embody the D-S-S sequence theoretically have the potential to promote tooth remineralization.

In a study by Zheng et al., the effect of polypeptides containing 8 D-S-S sequences (termed 8DSS) was assessed in a rat model of tooth decay [34]. The repeating sequences of carboxylates and phosphate groups of the peptide on either side of the 8DSS chain effectively interact with calcium ions. This interaction enhances the incidence of calcium-mediated bridging between the peptide chain and the surface of hydroxyapatite. The 8DSS peptides not only prevent the depletion of calcium and phosphate ions from the tooth enamel but also promote the incorporation of these ions from the surrounding medium into new mineral deposits on the enamel surface. However, Zheng et al. did not investigate the mechanical strength of the remineralization induced by 8DSS. Thus, it remains unclear whether the remineralized layer formed by 8DSS can withstand the rigors of oral mastication.

In another study, Ding et al. synthesized a peptide derived from tuftelin (TDP) and tested its ability to bind to hydroxyapatite [35]. TDP, a non-amelogenin protein, is primarily deposited at the dentin–enamel junction. Due to its negative surface charge, TDP can draw upon numerous mineral ions at the dentin–enamel junction to stimulate and control the initial mineralization of enamel. Through in vitro pH cycling models, TDP has demonstrated its potential to induce remineralization and significantly augment the surface hardness of tooth enamel lesions.

Bacteria are the principal contributors to recurrent tooth decay. In an attempt to amplify the antibacterial capabilities of self-assembled peptides, Ren et al. synthesized QP5, a peptide derived from amelogenin (ADP). They mixed it with chitosan to formulate a hydrogel (CS-QP5) [36]. Chitosan (CS) carries a positive charge, enabling it to adhere to bacterial walls, thereby yielding a bacteriostatic effect. The antibacterial efficacy of the CS-QP5 hydrogel is impressive, reaching up to 95.43%—the effectiveness peaks at 100% with an increased CS concentration (2.5 mg/mL).

Under acidic conditions, such as a pH of 4.5, almost all the amino groups in CS are expected to be completely protonated, which allows them to trap hydrogen ions and elevate the pH. The electrostatic interaction allows CS to engage with QP5, preventing QP5 from dissolving. As bacterial activity is inhibited and acidity decreases, CS weakens its bond with QP5, thereby releasing QP5 to attract calcium and phosphate ions from the environment and stimulate tooth enamel remineralization.

CS-QP5 has exhibited its dual benefits of antimicrobial activity and remineralization in laboratory settings. Nonetheless, considerations regarding the safety of CS in live organisms should not be overlooked, despite carboxymethyl chitosan’s potential to mitigate the side effects of CS [88].

Synthetic peptides primarily serve two functions. One of these functions is performed by antimicrobial peptides (AMP), such as IBM-1, IBM-2, and KSL (KKVVFKVKFK–NH2, a novel antimicrobial decapeptide derived using synthetic combinatorial library technology), which display potent antibacterial activity against drug-resistant bacteria [89,90]. The other function is remineralization, facilitated by peptides like P11-4, OSN, and 8DSS, which encourage mineral deposition and the repair of crystal structure [35,91].

Wang et al. engineered an α-helical AMP known as GH12 and the hydrophilic C-terminal “tail” of amelogenin referred to as TD7 [37]. TD7 has been observed to bond with calcium ions and stabilize hydroxyapatite (HA) structures [16]. Wang et al. combined the functional elements of GH12 and TD7 to create peptides with both antibacterial and remineralizing properties. These peptides have demonstrated an ability to inhibit biofilm formation within 24 h in vitro, resulting in shallower depths of lesions and increased mineral content. However, the process of synthesizing such multifunctional peptides is intricate.

Sarah et al. introduced metallic silver to amelogenin-derived peptides (ADP) and attached it to the amorphous calcium phosphate (ACP) nanocomplex [92]. They exploited the antibacterial capability of silver ions and ACP’s remineralization ability to prompt the rapid remineralization of silver diamine fluoride (SDF)-treated teeth.

Although the ability of self-assembled peptides to induce remineralization in vitro has been demonstrated, the preparation method is complex. Therefore, further enhancements are needed to achieve remineralization and antimicrobial effects concurrently. Moreover, the influence of these self-assembled peptides has been observed in in vitro caries models. However, it remains to be determined whether they can also maintain the physicochemical attributes of teeth under in vivo conditions.

## 7. Resin-Based Material

Resin-based sealants have been employed for sealing cracks for decades, significantly contributing to preventing dental caries through physical sealing methods [93]. Recently, numerous bioactive resin binders have emerged with capabilities such as tooth remineralization, antibacterial properties, and self-repairing cracks. However, resin-based adhesives face significant challenges, including microcracks at the edges caused by polymerization shrinkage, cyclic loading, and thermal and mechanical fatigue [94].

Amorphous calcium phosphate (ACP) can enhance the pH at the repair interface, release ions, and improve the remineralization effect of resin binders when incorporated into them [59]. Poly(urea-formaldehyde) (PUF) has demonstrated potential for self-repairing cracks [95]. Yue et al. developed a resin binder with antibacterial, self-healing, and remineralization properties using PUF, dimethylaminohexadecyl methacrylate (DMAHDM), and ACP [38]. DMAHDM is a positively charged monomer that can rupture bacteria by interacting with the negatively charged cell membranes [96]. Research has confirmed that an adhesive consisting of ACP and DMAHDM can enhance the concentration of Ca and P ions in biofilms, neutralize bacterial acids, exterminate bacteria, and enable the remineralization of dentin lesions [97].

Yue et al. incorporated 7.5% PUF in the form of 70 μm microplastic granules into the adhesive along with 10% DMAHDM and 20% ACP [38]. The adhesive layer between the resin adhesive and dentin was approximately 110 ± 20 μm, and the adhesion strength to dentin was not compromised. Resin binders with added PUF achieved a healing efficiency of 67%. In vitro experiments showed that they inhibited 95% of bacteria in biofilms and reduced the production of lactic acid by bacteria.

The polymerization shrinkage of dental composites can result in issues like the formation of enamel cracks, gaps both inside and on the edges, and a decrease in bond strength [98]. This shrinkage can leave dentin tubules vulnerable to bacterial infiltration, leading to failure of the adhesive [99]. Resin materials with reduced polymerization shrinkage can counteract bacterial invasion at the dental restoration interface [100]. Nevertheless, lower polymerization shrinkage can significantly impact the adhesive strength of resin materials.

Ghalia et al. engineered a substance known as triethylene glycol divinylbenzyl ether (TEG-DVBE) [39]. When urethane dimethacrylate (UDMA) is incorporated into TEG-DVBE, it markedly enhances conversion and intensity without a corresponding increase in polymerization shrinkage. Moreover, including non-collagenous proteins (NCP) can augment the remineralization potential of resin materials. Research has demonstrated that the polymerization shrinkage of this composite resin is 2.48 ± 0.03 Mpa, a value 36% less than resin without UDMA. Critically, adding UDMA does not compromise the mechanical properties of the composite. In vitro, antibacterial tests have confirmed that adding UDMA to the composite resin does not impact its antibacterial efficacy for up to 90 days. Compared to commercially available fluoride, this resin compound demonstrated the ability to remineralize dentin margins, increasing the hardness of dentin by up to 41%.

Calcium silicate resin demonstrates superior shear bond strength compared to traditional self-adhesive resins, effectively sealing dentin tubules [101]. When enhanced with calcium and phosphate ions, adhesive resins can function as ionic reserves, fostering the remineralization of tooth surfaces. Gabriel et al. formulated bioactive glass (BAG) in nanoparticles with sizes ranging from 0.05–2 μm and integrated them into a binder [40]. Their findings suggested that BAG-containing adhesive resins could maintain a higher hardness level for 28 days. Despite the increased hardness of the composite resins, Gabriel et al. did not assess them for their remineralization and antimicrobial capabilities.

Heba et al. incorporated calcium fluoride into ACP + DMAHDM resin to create a composite resin exhibiting remineralization and anti-biofilm properties [41]. This composite resin releases significant amounts of fluoride and calcium ions, inducing remineralization. The flexural strength of this new composite reached 125.93 ± 7.49 MPa, aligning with the recommended ISO range.

**Figure 3:**
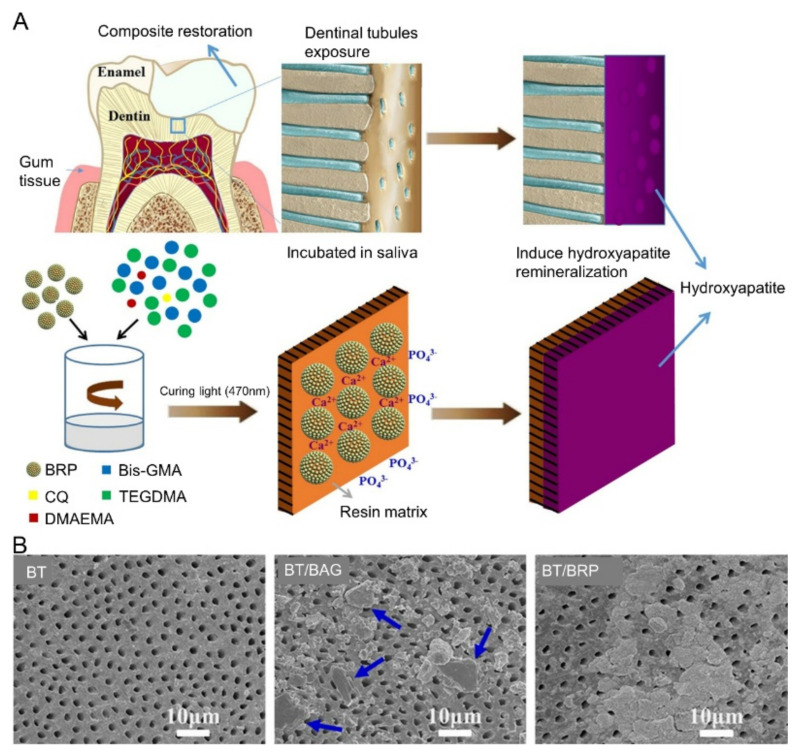
(**A**) Schematic demonstration of the specific mix of BRP and resin matrix and the subsequent in situ remineralization of HA. (**B**) SEM image of dentin remineralization induced by the three composites (BT, BT/BG, and BT/BRP) after immersion in AS for 30 days (the arrows point to the BAG particles). Reproduced with permission from [42].

Nonetheless, the hydrophilic nature of bioactive glass (BAG) fillers presents a compatibility issue with hydrophobic resin matrices. They have a tendency to cluster within the resin matrix, often causing a significant decline in the resin’s mechanical properties [102]. To address this, Li et al. modified BAG to enhance its hydrophobicity and partially incorporated it into bioactive amphiphilic raspberry-like composite nanoparticles (BRPs) to form resin composite particles (Figure 3A) [42]. BRPs have a diameter of 100 nanometers. A substantial remineralization depth can be attained with 63% coverage of dentin tubules by these resin composite particles (Figure 3B). As demonstrated in in vitro models, BRP-infused resin composites have improved mechanical attributes, water absorption resistance, and solubility due to their amphiphilic surface characteristics.

## 8. Synthetic Polymers

Despite the substantial therapeutic achievements of resin materials as sealants, they fundamentally differ from enamel. Over time, resin sealants are prone to a certain extent of micro-leakage [103]. The perfect sealant would combine oral-chewing mechanical strength with excellent penetration properties to prevent micro-leakage between the material and natural enamel [104].

**Figure 4:**
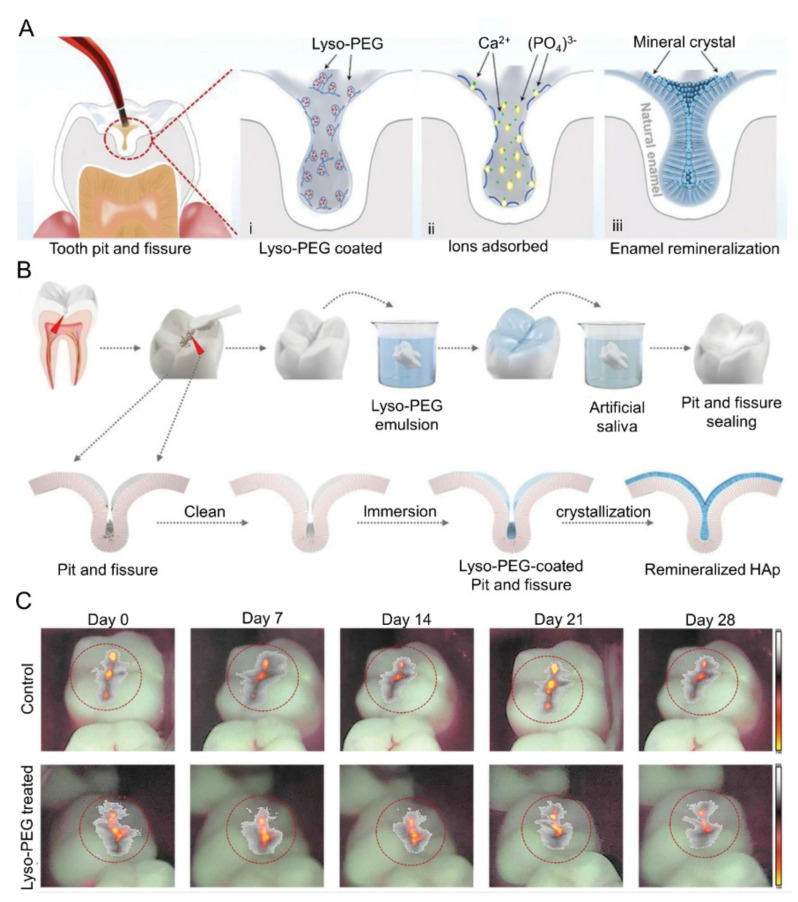
(**A**) Schematic of the lyso-PEG-induced remineralization process in pits and fissures. (**B**) Schematic diagram of remineralization within the lyso-PEG-coated pits and fissures. (**C**) QLF images of the second pits and fissures of the mandibular molar. The red circle refers to changes in fluorescence intensity of plaque biofilm adhesion in the pit and fissure areas. Reproduced with permission from [43].

Yang et al. tethered the human lysozyme with polyethylene glycol (PEG) to create lysozyme-PEG, which forms amyloid oligomer nanoparticles in an aqueous solution (Figure 4A) [43]. Lysozyme-PEG has the advantage of directly promoting the growth of the hydroxyapatite (HAp) crystal layer within fractures, boasting robust interfacial bonding stability and antibacterial capabilities. It also demonstrates excellent permeability, penetrating up to 20,000 μm within 180 s, and forms a nanomembrane at the infiltrated depth to trigger remineralization (Figure 4B).

The hardness and elastic modulus of the newly formed remineralized layer are comparable to those of natural enamel. Furthermore, lysozyme-PEG shows commendable biocompatibility, with its usage in the human body not leading to an increase in inflammatory factors (TNF-α, IL-6, and IL-1β). Clinical studies suggest that lysozyme-PEG, which can be applied or used as a gargle (Figure 4C), shows promising potential for deep adhesion and remineralization, surpassing traditional sealants.

Alendronate sodium (ALN), which contains two phosphate groups, has a high affinity for hydroxyapatite (HA) [105]. ALN, widely used in treating osteoporosis and other hard-tissue-targeted drug delivery systems, inhibits osteoclast activity [106]. In a study by Xu et al., ALN was grafted onto polyacrylic acid (PAA) to create ALN-PAA, which simulates non-collagenous proteins (NCP) [44]. After substituting zinc into HAs (ZHA), ZHA@ALN-PAA was formed on the outermost mineralized ZHA of ALN-PAA. Given ALN’s strong binding to tooth enamel, ALN-PAA aids in the absorption and release to stimulate remineralization, while zinc provides antibacterial benefits. In vitro, through pH alternations in simulated oral environments, ZHA@ALN-PAA forms nanorod structures that effectively fill gaps to promote remineralization.

Furthermore, Xu et al. developed a micelle named PMs using 3-maleimidopropionic acid-poly (ethylene glycol)-block-poly(l-lysine)/phenylboronic acid (MAL-PEG-B-PLL/PBA) [45]. A peptide isolated from oral saliva species, referred to as SAP, along with sodium fluoride, was combined with PMs to create a multifunctional nanosystem known as PMs@NaF-SAP. This nanosystem leverages PEG’s amphoteric properties for biofilm penetration and antibacterial action, uses SAP for HA-bound tooth surface adhesion, and releases NaF for tooth remineralization. PMs@NaF-SAP can adjust its contents according to changes in body pH to provide antimicrobial and remineralization effects. It has been demonstrated that this approach holds clinical translation potential in rodent caries models.

## 9. Conclusions and Outlook

With significant strides in biology and biomaterials development, curing dental caries, a previously challenging issue, appears achievable in the future. In both in vivo and in vitro studies, bioactive materials have demonstrated considerable potential in terms of dentin remineralization and antibacterial properties. Each bioactive material—fluoride, amorphous calcium phosphate (ACP), bioactive glass, collagen, self-assembling peptides, resin-based materials, and synthetic polymers—exhibits distinct strengths and weaknesses in terms of its antibacterial and remineralization effects. However, advancements in these substances can sufficiently offset their inherent limitations.

While synthetic polymer materials meet the requirements for dentin remineralization and antibacterial effects and offer hardness and elasticity comparable to natural tooth enamel, only a few have made it to clinical practice. As such, the real-world application of bioactive restorative polymer materials may present more complexities than initially anticipated.

Future research and development of bioactive materials should principally address the following issues:

1. The filling must establish complete adhesion to the tooth surface to prevent leakage.
2. The filling must possess adequate compressive strength to withstand the forces exerted during mastication.
3. The filling must be biocompatible and not pose toxicity risks to the surrounding tooth tissues.

We strongly believe that the utilization of biomaterials in dental caries treatment will eventually yield impressive clinical outcomes.

## Figures and Tables

**Table 1:** Biomaterials trigger remineralization and antimicrobial effects for dental caries restoration.

Type	Name	Element	Characteristic	Advantage	Disadvantage	Result	Reference
Fluoride	Arg-NaF toothpaste	Arginine, NaF	Arginine: NaF = 1:3	Increase the concentration of fluoride ions in clean toothpaste	As the arginine concentration increases, the pH of the toothpaste gradually decreases	NaF toothpaste significantly increased the remineralization of incipient enamel caries	[20]
	Polyhexamethylene biguanide-AgF	Polyhexamethylene biguanide, AgF	PHMB-F has stronger antibacterial activity	PHMB-F has stronger antibacterial activity	The long-term safety of PHMB-F in vivo is unknown	PHMB-F has good antibacterial and remineralization capabilities	[21]
Amorphous calcium phosphate	Gluey silver–calcium phosphate (GSCP)	GSCP, ACP	GSCP stabilizing the ACP phase and providing binding sites for Ag ions	GSCP releasing calcium ions and phosphates provides the basis for remineralization	GSCP is a jelly-like structure that does not adhere stably to the tooth’s surface	GSCP has good antibacterial and remineralization capabilities	[22]
	NACP binder nanoparticle	Binder, ACP	The average particle size of NACP was 116 nm	The ACP binder-treated enamel showed the best remineralization	The released calcium and phosphorus ions are only effective in situ.	The ACP binder showed a remineralization value of 52.29 ± 4.79%	[23]
	CPP-ACP	CPP, ACP, Sodium trimetaphosphate	-	CPP-ACP decrease in mineral loss on the surface of the teeth	-	CPP-ACP can reduce the ability to demineralize teeth	[24]
	CPP-ACP -SnF2	Stannous fluoride, CPP, ACP	SnF2 is remineralized by substituting hydroxyl groups in hydroxyapatite	SnF2 also enhances the ability of the mixture to release fluoride ions	-	SnF2+CPP-ACP could increase the degree of remineralization by 32% on the tenth	[25]
	PCBAA/ACP nanocomposites	PCBAA, ACP	PCBAA/ACP nanocomposite has an average particle size of 50.67 ± 2.37 nm	PCBAA/ACP nanocomposites can efficiently enter the dentin tubules for deep remineralization.	-	PCBAA/ACP nanocomposites has good antibacterial and remineralization capabilities	[26]
	45S5 BAG	45S5 BAG	BAG is mainly embedded in the dentin tubules to form deposits	Compared with the CPP-ACP, BAG can lead to deep remineralization	-	BAG has good remineralization capabilities	[27]
	BAG-silver oxide	BAG, silver oxide	The average particle size of BAG is about 4–5 μm	Silver oxide promotes the release of more cations	Silver oxide reduces the release of silicon ions.	BAG-silver oxide has good remineralization	[28]
	PRG	calcium fluoride, pre-reacted glass-ionomer (PRG)	The average diameter of PRG was 4.89 μm	PRG has calcium ions released and the stronger the remineralization ability	High calcium fluoride concentrations will affect composites’ physical and chemical properties	PRG enhance BAG’s remineralization capacity	[29]
Collagen	PAMAM-MMP inhibitors peptide	PAMAM, Galardin	The average diameter of PAMAM-peptide was 16.8 nm	The carboxyl groups in PAMAM attract ACP to be deposited on the collagen scaffold	-	PAMAM-peptide can effectively fight dentin caries in rats	[30]
	ACP -PAMAM	PAMAM, ACP	-	PAMAM/ACP has stronger the remineralization		PAMAM/ACP showed superior remineralization capacity of human dentin type I collagen fibers	[31]
	Succinic acid -modified collagen	Succinic acid, collagen fiber		SA-modified collagen fiber scaffolds can increase the mineralization rate	SA-modified collagen did not significantly improve the mechanical properties	SA-modified collagen fiber scaffolds can increase the mineralization rate	[32]
	Cross-linking collagen	Tannic acid, collagen	The degree of cross-linking of TA to collagen was 41.28 ± 1.52	The polymer enhanced its resistance to collagenase	The self-assembly process of TA and polymer is affected by pH	Cross-linking collagen has good remineralization	[33]
Self-assembling peptides	8DSS	8DSS	8DSS chain can interact effectively with Ca2+	8DSS chain can interact effectively with Ca2+	The remineralized layer formed by 8DSS meets the hardness of oral chewing is unknown	8DSS peptides prevent the leaching of calcium and phosphate ions	[34]
	Tuftelin-derived peptide	Tuftelin-derived peptide	-	TDP is a non-amelogenin protein that is deposited mainly at the dentin-enamel junction.	-	TDP has been shown to have the ability to induce remineralizatio	[35]
	Chitosan-QP5 (an amelogenin-derived peptide)	Chitosan, QP5	The inhibition rate of CS-QP5 hydrogel is as high as 95.43%	CS-QP5 has demonstrated its dual antimicrobial and remineralizing effects in vitro	the safety of CS in vivo is unknown	Chitosan-QP5 has been shown to have the ability to induce remineralizatio	[36]
	TD7	-	TD7 has been shown to bind to calcium ions and stabilize HA vigilance structures	TD7 has been shown to bind to calcium ions and stabilize HA vigilance structures	-	TD7 peptides effectively inhibit biofilm formation, shallower lesion depth, and higher mineral content.	[37]
Resin-based material	PUF-DMAHDM-ACP resin binder	PUF, DMAHDM, ACP	7.5% PUF in 70 μm microplastic granules and added them to the adhesive together with 10% DMAHDM and 20% ACP	PUF-DMAHDM-ACP resin binder can neutralize bacterial acids, kill bacteria, and achieve remineralization		PUF-DMAHDM-ACP resin binder inhibit 95% of bacteria in biofilms and reduce the amount of lactic acid	[38]
	TEG-DVBE- UDMA	TEG, DVBE, UDMA	UDMA added to TEG-DVBE increases conversion and intensity	UDMP did not affect the mechanical properties of the composite		TEG-DVBE- UDMA increasing dentin hardness by up to 41% compared to commercial fluoride	[39]
	BAG nanoparticles resin	BAG, 0.05–2 μm nanoparticles, calcium silicate resin	BAG synthesized into 0.05–2 μm nanoparticles and incorporated them into a calcium silicate resin	composite resins for remineralization and antimicrobial power		BAG nanoparticles resin withstand the challenge of higher hardness for 28 days.	[40]
	calcium fluoride- ACP-DMAHDM resin	Calcium, ACP, DMAHDM resin	BAG fillers are hydrophilic and incompatible with hydrophobic resin matrices	calcium fluoride- ACP-DMAHDM resin releases high fluoride and calcium ions to induce remineralization	BAG fillers tend to aggregate in the resin matrix, may lead to a serious deterioration in the mechanical properties	calcium fluoride- ACP-DMAHDM resin flexural strength was 125.93 ± 7.49 MPa, within the recommended range of ISO	[41]
	BRP-containing resin composites	BAG, BRPs	BRP has a diameter of 100 nanometers resin composites has amphiphilic surface properties	BRP-containing resin composite particles have good mechanical properties, water adsorption resistance, and solubility		BRP-containing resin composites have better mechanical properties, water adsorption resistance, and solubility	[42]
Synthetic polymers	lysozyme-PEG nanoparticles	Lysozyme, polyethylene glycol (PEG)	lysozyme-PEG nanoparticles penetrate to 20,000 μm in 180 s and form a nanomembrane	lysozyme-PEG has strong interfacial bonding stability, antibacterial ability and good biocompatibility		lysozyme-PEG has deep adhesion and remineralization with simple application or gargle	[43]
	ZHA@ALN-PAA	PAA, ALN, ZHA	simulate NCP	ZHA@ALN-PAA can adsorbs and releases phosphate ions to promote remineralization		ZHA@ALN-PAA can simulated oral environments and form nanorod structures for remineralization	[44]
	PMs@NaF-SAP	PM3, SAP, NaF	PMs@NaF-SAP has antibacterial effect and remineralization ability	PMs@NaF-SAP can remove contents to achieve antimicrobial and remineralization effects		PMs@NaF-SAP has clinical translational potiential in redent caries models	[45]

## Data Availability

Not applicable.

## Sample Availability

Not available.
